# Expert Consensus on the Management of Obesity in Older Adults (2026 Edition)

**DOI:** 10.1002/agm2.70105

**Published:** 2026-09-02

**Authors:** Qi Pan, Lixin Guo

**Affiliations:** ^1^ Department of Endocrinology Beijing Hospital, National Center for Gerontology Beijing China; ^2^ National Clinical Research Center for Gerontology Beijing China; ^3^ The Key Laboratory of Geriatrics of NHC Institute of Geriatric Medicine, Chinese Academy of Medical Sciences Beijing China

**Keywords:** body fat percentage, consensus, obesity, older adults, sarcopenia

## Abstract

Obesity in older adults has become a prominent public health issue. Owing to age‐related physiological changes and multimorbidity, obesity in this population is associated with complex clinical manifestations and markedly increased difficulty in management. Based on this, a multidisciplinary panel of obesity experts from Beijing Hospital, National Center for Gerontology, formulated this consensus after rigorous evaluation and iterative revision. The consensus emphasizes that management of obesity in older adults requires comprehensive assessment according to age and overall health status and should adopt a patient‐centered, individualized, and stratified strategy. By adhering to the principles of safety first, functional improvement, and steady progress, the goals are to reduce visceral fat accumulation, maintain or increase muscle mass, protect bone health, and improve physical function and overall health. Special attention should be paid to sarcopenia in older adults. Adequate intake of high‐quality protein, combined with an individualized resistance exercise program, is an effective strategy for maintaining muscle mass. Selection of weight‐loss medication should be guided by evidence supporting its benefit for obesity‐related complications or comorbidities, with close monitoring of adverse reactions.

## Graphical abstract

This graphical abstract summarizes a safety‐first framework for managing obesity in older adults, with attention to age, functional status, comorbidities, and sarcopenia risk. Key assessments include BMI, waist measures, body fat percentage, skeletal muscle mass, and comprehensive geriatric evaluation. Management prioritizes reducing visceral fat while preserving muscle and bone health through individualized nutrition, exercise, medication selection, behavioral support, and follow‐up. The goal is to improve physical function, reduce complications, and support safe, sustainable long‐term health in older adults with obesity. ASMI, appendicular skeletal muscle mass index; BFP, body fat percentage; BMI, body mass index; CGA, comprehensive geriatric assessment; WC, waist circumference; WHR, waist‐to‐hip ratio.

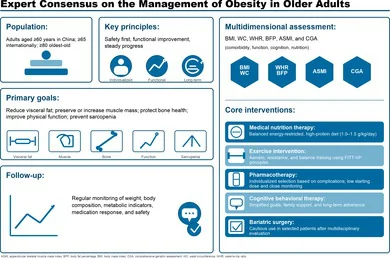

## 1 Introduction

Socioeconomic development and rapid population aging have resulted in obesity in older adults becoming an important public health issue. Obesity aggravates multisystem structural and functional impairment and seriously affects quality of life, and increases the economic burden on individuals, families, and society. Obesity in older adults, compared with younger people, has unique pathophysiological characteristics and clinical manifestations. Age‐related metabolic changes make it easier to accumulate fat, especially visceral and intramuscular fat, which interact with insulin resistance to impose a “double hit” on health [1]. In addition, aging‐related muscle loss, organ dysfunction, cognitive decline, and osteoporosis overlap with obesity and further increase health risks [2].

Management of obesity in older adults must take into consideration the rationale for and necessity of weight loss under different functional states, while also focusing on how to avoid muscle loss during weight reduction. Comprehensive assessment of physical function, cognitive status, sarcopenia, obesity‐related complications, and comorbidities, followed by patient‐centered and individualized stratified management, is essential for achieving an appropriate risk–benefit balance. The Expert Consensus on the Management of Obesity in Older Adults (2026 Edition) was developed to standardize clinical practice, improve the health of older adults, and reduce disease burden.

## 2 Part I Consensus Development Methodology

### 2.1 Process

This consensus was initiated by Beijing Hospital, National Center for Gerontology in July 2025 and finalized in April 2026. It strictly followed the latest Institute of Medicine definition of clinical practice guidelines and the methodology in the WHO Handbook for Guideline Development. It also referred to the Chinese Guideline for Developing/Updating Clinical Practice Guidelines (2022 Edition) and the development process described in Evidence‐Based Clinical Practice Guideline Development and Implementation. The protocol and full consensus were prepared according to the Scientific, Transparent and Applicable Rankings checklist and the Reporting Items for Practice Guidelines in Health Care. A protocol was developed and registered on the Practice Guideline Registration for Transparency platform (http://guidelinesregistry.cn/; PREPARE‐2025CN1576).

### 2.2 Target Population and Users

The target population of this consensus is older adults with obesity. It is intended for healthcare professionals in endocrinology and metabolism, geriatrics, general practice, clinical nutrition, and related disciplines in healthcare institutions at all levels for reference in patient diagnosis, treatment, and management.

### 2.3 Working Group

The core expert group included guiding experts, principal authors, and the secretariat. A consensus expert committee with regional and professional representation was established, led by the core experts. The committee included multidisciplinary experts in endocrinology and metabolism, geriatrics, surgery, sports medicine, clinical nutrition, and rehabilitation medicine. All participating experts completed conflict‐of‐interest declarations, which were disclosed and managed according to relevant requirements to ensure independence and objectivity in the consensus development process.

### 2.4 Clinical Question Identification

The working group systematically reviewed domestic and international literature, guidelines, and expert consensuses on the diagnosis, treatment, and management of obesity in older adults. Candidate clinical questions were developed based on clinical practice experience and the Population, Intervention, Comparison, Outcome (PICO) framework. One round of a modified Delphi questionnaire was conducted using a 5‐point Likert scale to rate the importance of all candidate clinical questions (1 = least important, 5 = most important), and four key clinical questions were finalized.

### 2.5 Literature Search and Evaluation

The working group searched PubMed, the Cochrane Library, the Guidelines International Network (GIN), and the Wanfang Medical Database for evidence on the epidemiological characteristics, screening and diagnosis, and comprehensive management of obesity in older adults. The search period was January 2000 to March 2026. Chinese keywords included “obesity,” “older adults,” “oldest‐old,” “sarcopenia,” “body mass index,” “waist circumference,” “waist‐to‐hip ratio,” “body fat percentage,” and “skeletal muscle mass index”; English keywords included “obesity,” “elderly or older,” “body mass index,” “sarcopenia,” “waist circumference,” “waist‐to‐hip ratio,” “body fat percentage,” and “skeletal muscle mass index.” Eligible evidence included randomized controlled trials, prospective cohort studies, retrospective studies, systematic reviews, meta‐analyses, guidelines, and expert consensuses.

### 2.6 Formation of Recommendations

The Consensus Expert Committee integrated evidence‐based medical evidence, China's medical policy system, cost‐effectiveness, and other key factors. After three rounds of online and offline consensus meetings, expert committee members voted on each item. A consensus was reached when the proportion of experts agreeing exceeded 80%, and eight key points were ultimately formed.

## 3 Part II Epidemiology and Hazards

### 3.1 Epidemiological Characteristics

Internationally, people aged ≥ 65 years are considered older adults, whereas in China, people aged ≥ 60 years are defined as older adults and those ≥ 80 years as the oldest‐old. According to the 2024 Statistical Bulletin on the Development of Civil Affairs issued by the Ministry of Civil Affairs of China, by the end of 2024, China had 310 million people aged ≥ 60 years, accounting for 22.0% of the total population, and 220 million people aged ≥ 65 years, accounting for 15.6% [3]. The prevalence of obesity generally increases with age but decreases in later life; possibly because of increased sarcopenia and survival bias among older adults. A meta‐analysis showed that the average global prevalence of obesity (body mass index [BMI] ≥ 30 kg/m^2^) in older adults was 25.3%; lowest in Asia (14.6%) and highest in South America (40.4%) [4]. The Fifth National Physical Fitness Surveillance Bulletin reported that, in 2020, the prevalence of obesity (BMI ≥ 28 kg/m^2^) among Chinese adults aged ≥ 60 years was 16.7% and increasing [5]. Body fat percentage (BFP) is higher in women than in men. It remains stable in urban older adults, whereas it declines more obviously after age 60 years in rural older adults, especially in men.

Obesity in older adults is highly heterogeneous. A cross‐sectional survey of 15.8 million Chinese adults showed that the prevalence of obesity peaked at age 35–39 years in men and at age 70–74 years in women [6]. The China Cardiometabolic Disease and Cancer Cohort study showed that the association between obesity and health outcomes varies by sex and age. The obesity threshold associated with the lowest all‐cause mortality risk was higher in men than in women, and a moderately higher BMI (25.0–27.1 kg/m^2^) may be beneficial for health outcomes in older adults [7]. Sarcopenic obesity (SO) is particularly common among older adults. Meta‐analyses reported a global prevalence of 9%–11% in older adults [8, 9] and 6%–7% in Chinese older adults [10, 11].

### 3.2 Health Hazards

Obesity is closely associated with increased risks of multisystem diseases in older adults. It causes type 2 diabetes mellitus (T2DM), metabolic dysfunction‐associated steatotic liver disease (MASLD), cognitive impairment, kidney disease, urinary incontinence, and malignancy, and accelerates the development of hypertension, dyslipidemia, and atherosclerotic cardiovascular disease (ASCVD). By increasing mechanical joint load, obesity increases the risk of knee osteoarthritis and osteoporotic fractures in older adults. The high prevalence of obstructive sleep apnea (OSA) in middle‐aged and older adults is closely related to obesity. SO is an increasingly prominent health issue. Compared with sarcopenia or obesity alone, the combination of muscle loss and obesity synergistically aggravates metabolic disorders in older adults, significantly increases cardiovascular and all‐cause mortality risks [12, 13], and is associated with higher risks of falls, frailty, and disability [14, 15].

### 3.3 Disease Burden

Obesity significantly increases the burden on older adults, their families, and society. In addition to physical health effects, obesity may cause depression and anxiety. Older adults with obesity may reduce social activities because of difficulty with physical activity, which can lead to loneliness and social isolation. Older adults with obesity are often exposed to polypharmacy, increasing the risk of adverse drug interactions. Obesity is closely associated with cognitive decline. It affects quality of life and imposes an additional caregiving burden on families and society. An American study found that medical expenditure was 6%–13% higher in older men with obesity than in normal‐weight men and 11%–17% higher in women with obesity [16]. Another study showed that, although obesity and its complications increase healthcare costs, the cost‐effectiveness of weight loss remains controversial because low body weight in older adults may be associated with higher mortality [17]. These findings suggest the need for more targeted intervention strategies to reduce disease burden in older adults with obesity.

## 4 Part III Diagnosis and Pretreatment Evaluation

Key Point 1: Screening and diagnosis of obesity in older adults should be based on multidimensional assessment including BMI, waist circumference (WC), waist‐to‐hip ratio (WHR), BFP, and muscle mass. If sarcopenia is suspected, muscle strength and physical function parameters related to skeletal muscle function should be further assessed.

Key Point 2: Before treating obesity, health status, etiology and severity of obesity, obesity‐related complications and comorbidities, and sarcopenia should be comprehensively assessed.

BMI is a commonly used indicator for screening for obesity, but it cannot distinguish fat from muscle mass and may therefore misclassify health risk, particularly in older adults. Older adults often experience height loss and reductions in muscle and bone mass, resulting in changes in body composition; thus, the same BMI may correspond to a higher BFP. Therefore, in addition to BMI, screening and diagnosis of obesity in older adults should incorporate WC, WHR, and measurement of BFP and muscle mass by bioelectrical impedance analysis (BIA) or dual‐energy X‐ray absorptiometry (DXA) for multidimensional assessment (Table 1). If sarcopenia is suspected, muscle strength and physical function parameters related to skeletal muscle function should be evaluated. Previous studies have suggested that waist‐to‐height ratio may be combined with BMI to assess obesity in older adults [18], but current data remain insufficient and further evidence is needed.

**TABLE 1:** Multidimensional assessment parameters for body weight in older adults.

Parameter	Reference range	Measurement	Clinical implication
BMI	22.0–26.9 kg/m^2^	Digital body weight scale and stadiometer (accuracy 0.1 kg and 0.1 cm)	Screening for overweight/obesity
WC	< 90 cm (men); < 85 cm (women)	Standing naturally with feet 25–30 cm apart. Locate the midpoint between the lower margin of the last palpable rib and the top of the iliac crest. Place a measuring tape horizontally around the abdomen at the level of the midpoint. Ensure the tape is snug but does not compress the skin	Identifying abdominal obesity, evaluating cardiovascular risk
WHR	< 0.90 (men); < 0.85 (women)	Ratio of waist‐to‐hip circumference. Measure hip circumference with legs together. Place the tape horizontally at the level of the pubic symphysis and the most protruding part of the gluteus maximus, and wrap it around the buttocks for one full circumference	Identifying abdominal obesity, evaluating cardiovascular risk
BFP	< 27.5% (men); < 33% (women)	BIA (screening), DXA (gold standard), CT/MRI when necessary (to measure visceral fat area)	Screening for overweight/obesity
Skeletal muscle mass	ASMI: ≥ 7.0 kg/m^2^ (men); ≥ 5.7 kg/m^2^ (women)	ASMI = appendicular skeletal muscle mass (kg)/height^2^ (m^2^). BIA (screening), DXA (muscle mass measurement error < 3%)	Evaluating metabolic capacity and cardiovascular risk, screening for sarcopenia

Abbreviations: ASMI, appendicular skeletal muscle mass index; BFP, body fat percentage; BIA, bioelectrical impedance analysis; BMI, body mass index; DXA, dual‐energy X‐ray absorptiometry; WC, waist circumference; WHR, waist‐to‐hip ratio.

### 4.1 Screening and Diagnosis

#### 4.1.1 BMI

BMI is a commonly used indicator for screening and diagnosis of generalized obesity and correlates well with BFP. According to the Guidelines for the Diagnosis and Treatment of Obesity (2024 Edition) [19], BMI of 18.5–23.9 kg/m^2^ is considered normal in Chinese adults, 24.0–27.9 kg/m^2^ indicates overweight, and ≥ 28 kg/m^2^ indicates obesity. With aging, height decreases, lean body mass declines, and BFP increases, which substantially limits the use of BMI to assess obesity in older adults. A prospective cohort study in China showed that among adults aged ≥ 65 years, all‐cause mortality risk was lowest at BMI of 25.0–26.9 kg/m^2^ and significantly increased at BMI < 22 kg/m^2^ and ≥ 30 kg/m^2^ [20]. The China Cardiometabolic Disease and Cancer Cohort study (mean age, 56.5 years) showed that all‐cause mortality risk was lowest at BMI of 25.0–27.1 kg/m^2^, and the association of increased BMI with cardiovascular disease and all‐cause mortality weakened with age. In the oldest‐old men, in particular, higher fat content may even be associated with lower cardiovascular disease and mortality risk [7]. Chinese adults aged ≥ 80 years have a low mean BMI (21.9 kg/m^2^), which decreases with age [21]. In the oldest‐old population, BMI has a U‐shaped association with all‐cause mortality and a linear negative association with the risk of impairment of activities of daily living (ADL) [22, 23]. In combination with the Appropriate Range of Body Mass Index and Weight Management Standard for the Oldest Old issued by the National Health Commission in July 2025 [24], this consensus recommends a BMI of 22.0–26.9 kg/m^2^ for adults aged ≥ 65 years. Maintenance of BMI within this range can reduce all‐cause, cardiovascular, and cancer mortality risks [25].

#### 4.1.2 WC and WHR

WC and WHR are commonly used indicators of central obesity and can be used to assess individual differences in body fat distribution and cardiometabolic risk. In Chinese adults, normal WC is defined as < 85 cm in men and < 80 cm in women; central obesity can be diagnosed at WC ≥ 90 cm in men and ≥ 85 cm in women. WHR ≥ 0.90 in men and ≥ 0.85 in women can also be diagnostic for central obesity [19]. A retrospective cohort study in Chinese older adults showed that an increase in WC, regardless of baseline measurement, was associated with increased cardiovascular mortality risk, suggesting the harmful effect of visceral fat on cardiovascular health in older adults [26]. A trajectory analysis of a large Chinese prospective cohort also found that high WC significantly increased future cardiovascular disease risk. The China Cardiometabolic Disease and Cancer Cohort study showed positive associations of WC and WHR with cardiovascular disease risk but U‐shaped associations with all‐cause mortality. Optimal WC and WHR based on all‐cause mortality risk differed by sex: WC 88 cm and WHR 0.90 in men, and WC 83 cm and WHR 0.85 in women [7]. Therefore, this consensus recommends the same reference standards for WC and WHR in older adults as in the general adult population. Combination of WHR with WC can better guide clinical practice. A Mendelian randomization study in the oldest‐old Chinese population showed that combination of higher BMI (28.0 kg/m^2^) and lower WC (75–80 cm) was associated with the lowest mortality risk. Subgroup analyses suggested a monotonic positive association between WC and mortality risk in men, whereas WC 80–90 cm did not further increase mortality risk in women [27].

#### 4.1.3 BFP

BFP can be measured using BIA, DXA, computed tomography, and magnetic resonance imaging. DXA is more accurate for measuring muscle mass, whereas BIA is simpler and more convenient, making it better suited for large‐scale screening and diagnostic use in community and hospital settings. In adults, BFP > 25% in men or > 30% in women indicates excess adiposity [19]. Studies of the optimal BFP values to predict cardiovascular or all‐cause mortality in older Chinese adults are lacking. A meta‐analysis found that a 10% increase in BFP was associated with an 11% higher risk of all‐cause mortality in adults. In adults aged > 60 years, there was no increase in all‐cause mortality and a trend toward reduced risk, suggesting that high fat mass may protect against all‐cause mortality in older adults [28]. This consensus recommends a BFP reference of < 27.5% for older men and < 33% for older women. For older adults with sarcopenia, these criteria may be relaxed, with specific determinations made based on muscle mass and overall health.

#### 4.1.4 Skeletal Muscle Mass Index

Reduced muscle strength is a risk factor for multisystem chronic diseases, falls, and cognitive impairment and is also a predictor of all‐cause mortality in older adults [29]. Good muscle mass can prevent decline in muscle strength. This consensus recommends incorporating muscle mass into routine screening in older adults with obesity. Muscle mass is commonly assessed using the appendicular skeletal muscle mass index (ASMI). The 2025 Asia–Oceania Consensus: Definitions and Diagnostic Criteria for Sarcopenic Obesity recommends ASMI as a diagnostic criterion for sarcopenia, with commonly used BIA cutoffs of < 7.0 kg/m^2^ in men and < 5.7 kg/m^2^ in women [30]. A 2025 Chinese study using muscle strength indicators related to maintenance of good physical function and vitality as outcome parameters reported BIA‐measured ASMI cutoffs of 13.55 kg/m^2^ in men and 11.25 kg/m^2^ in women, providing an important reference for prevention of sarcopenia in older adults [31].

### 4.2 Pretreatment Assessment

Before initiating obesity treatment, a comprehensive geriatric health‐status assessment should be performed, followed by further assessment of obesity etiology, obesity‐related complications and comorbidities, and sarcopenia (Table 2) [32].

**TABLE 2:** Comprehensive health‐status assessment in older adults.

Health status	Diseases, activity, cognitive function, and nutritional status
Good health	Has ≤ 2 chronic diseases excluding obesity (including stroke, hypertension, stage 1–3 chronic kidney disease, osteoarthritis, etc.), and no ADL impairment, ≤ 1 IADL impairment, no cognitive impairment, and good nutritional status
Intermediate health	Has ≥ 3 chronic diseases excluding obesity and/or any one of the following: (1) ≥ 2 IADL impairments; (2) mild cognitive impairment or early dementia; (3) risk of malnutrition
Poor health	Has any one of the following: (1) ≥ 1 chronic disease with limited treatment and reduced life expectancy (such as metastatic malignant tumors, pulmonary disease requiring oxygen therapy, end‐stage renal disease requiring dialysis, advanced heart failure); (2) ≥ 2 ADL impairments; (3) moderate or severe dementia; (4) malnutrition

Abbreviations: ADL, activities of daily living; IADL, instrumental activities of daily living.

#### 4.2.1 Comprehensive Geriatric Health‐Status Assessment

A comprehensive geriatric assessment should include diseases, activity, cognitive function, and nutritional status, and should guide treatment plans aimed at maintaining and improving health and functional status in older adults (Table 2). Common assessment tools include ADL and instrumental ADL scales for basic self‐care and instrumental abilities; the Mini‐Mental State Examination for cognitive function; and the Mini Nutritional Assessment‐Short Form, a widely used nutritional screening tool that can identify potential nutritional problems in older adults and guide dietary intervention strategies for patients with obesity.

#### 4.2.2 Etiological Assessment

For individuals with obesity, the following should be obtained: comprehensive medical history, including past medical and family history; current medication; smoking and alcohol consumption; social history; previous weight‐loss attempts; weight‐change history and causes of weight gain; dietary and food intake patterns; exercise habits and intensity; and mental health status. Measurement of vital signs, a full physical examination, and laboratory tests should also be performed. In older adults, secondary obesity may result from: (1) endocrine diseases such as Cushing's syndrome, hypothyroidism, and adult growth hormone deficiency; and (2) drug‐induced obesity from glucocorticoids, antipsychotics, and antidiabetic drugs that promote weight gain.

#### 4.2.3 Assessment of Complications and Comorbidities

Obesity‐related complications fall into two main categories [33]. Fat mass diseases result from mechanical changes due to increased fat mass (e.g., OSA, knee osteoarthritis, and stress urinary incontinence). Sick fat diseases arise from metabolic, endocrine, inflammatory, and immune dysregulation, including T2DM, hypertension, dyslipidemia, CVD, heart failure, and MASLD. These conditions may precede obesity or coexist with it as primary diseases. For individuals without complications, weight loss should be the primary goal to reduce future risk. For those with complications or comorbidities, management should prioritize treating these conditions, and medication choice should be based on evidence of benefit.

#### 4.2.4 Assessment of SO

Some older adults have both sarcopenia and obesity, which is known as SO. Screening, diagnosis, and treatment can follow The 2025 Asia–Oceania Consensus: Definitions and Diagnostic Criteria for Sarcopenic Obesity [30]. Given limited evidence that weight loss benefits the oldest old, interventions in this group should be undertaken with particular caution.

## 5 Part IV Weight Management Principles and Goals

Key Point 3: Management of obesity in older adults should use a patient‐centered, individualized, and stratified strategy, with goals of reducing visceral fat accumulation, maintaining or increasing muscle mass, protecting bone health, and improving physical function and health status.

Key Point 4: Long‐term weight management should follow the principles of safety first, functional improvement, and steady progress to avoid weight regain and loss of muscle and bone mass.

**FIGURE 1:**
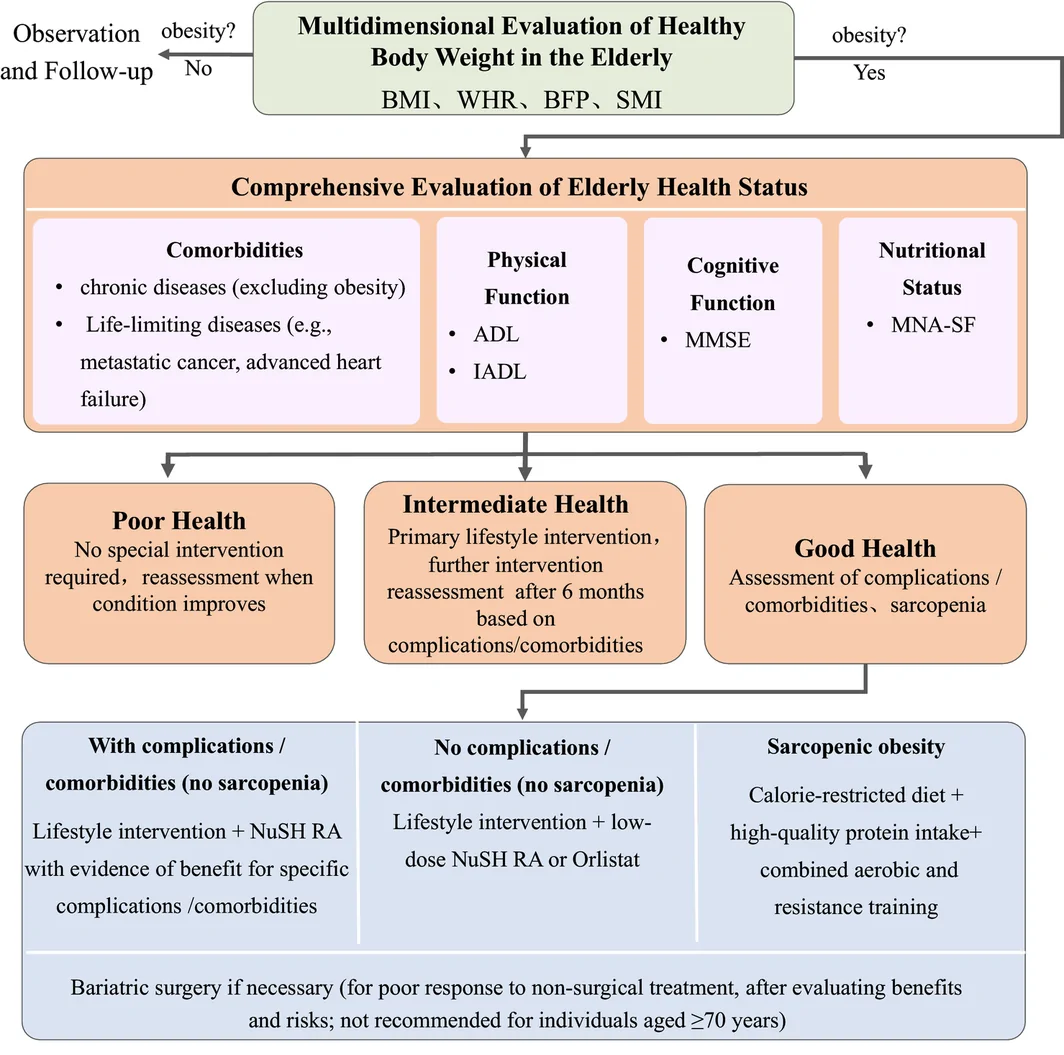
Pathway of assessment and intervention in obesity in older adults.

Obesity management in older adults should target reduced visceral fat, preserved or increased muscle mass, protected bone health, and improved physical function and overall health. Treatment planning should be based on a comprehensive assessment of health and cognitive status, sarcopenia, complications, and comorbidities. After carefully weighing the risks and benefits of weight loss, a patient‐centered, individualized, and risk‐stratified management strategy should be implemented (Figure 1).

Weight‐loss goals for older adults with obesity should follow the principles of “safety first, functional improvement, and steady progress to prevent weight regain and avoid loss of muscle and bone mass.” In young and middle‐aged adults with obesity, a 3%–5% weight reduction usually improves metabolic indicators such as blood glucose, blood pressure, and triglycerides and may be considered a clinically meaningful threshold. Therefore, older adults with obesity and good health status may set a further goal of 5%–10% weight loss within 1 year [34, 35, 36, 37] to reduce the risk of cardiometabolic disease. For those with intermediate health status, management should be oriented toward functional improvement with moderate weight loss. For those with poor health status and for the oldest‐old (aged ≥ 80 years), the goal should be weight stability. Clinicians should be particularly alert to rapid unintentional weight loss over a short period in older adults, which may indicate underlying disease.

The optimal weight‐loss regimen for older adults with obesity is centered on a healthy lifestyle, including professional dietary weight‐loss plans and regular physical exercise [38]. When patients have T2DM, ASCVD, chronic kidney disease (CKD), MASLD, OSA, knee osteoarthritis, or other diseases, etiological and symptomatic treatment of the primary disease is recommended, combined with weight‐loss medication supported by evidence of benefit for these diseases. In a small number of selected patients (aged < 70 years, in good health status, and with poor response to nonsurgical treatment), bariatric surgery may be considered after assessment of risks and benefits. Weight loss is not recommended in patients with consumptive conditions such as severe gastrointestinal disease, infection, malnutrition, or malignant tumors. During weight reduction in older adults, special attention should be paid to loss of muscle and bone mass. Whether from calorie‐restricted diet intervention or weight‐loss medications, 25%–45% of total weight loss comes from lean mass loss, increasing the risk of muscle loss [39, 40]. Ensuring adequate high‐quality protein intake and combining exercise intervention, especially resistance training, can maintain or even increase muscle mass, and is an effective strategy for reducing obesity‐related cardiovascular events [41, 42, 43].

## 6 Part V Intervention

Key Point 5: The optimal weight‐loss regimen is centered on a healthy lifestyle, including professional dietary weight‐loss plans and regular physical exercise.

Key Point 6: Medical nutrition therapy should use a balanced, calorie‐restricted, high‐protein diet (1.0–1.5 g/kg/day); whey protein and β‐hydroxy‐β‐methylbutyrate (HMB) may be supplemented when necessary.

Key Point 7: Exercise prescriptions should be based on the FITT‐VP framework (frequency, intensity, type, time, volume, and progression) and the principles of individualization, safety, and synergy, combining aerobic exercise, resistance training, and balance exercises.

Key Point 8: Weight‐loss medication with evidence of benefit should be selected according to complications or comorbidities, with etiological and symptomatic treatment of comorbidities; weight‐loss medications should be started at a low dose with close monitoring of adverse reactions.

### 6.1 Medical Nutrition Therapy

Several studies have used balanced calorie‐restricted diets with regular foods to create a daily energy deficit of 500–750 kcal, achieving 5%–10% weight loss within 6–18 months [44, 45]. For older adults with obesity, this consensus recommends a balanced calorie‐restricted diet (very‐low‐calorie diets are not recommended) with appropriately increased protein intake. In those with normal renal function, protein intake should be 1.0–1.2 g/kg/day and may be increased to 1.5 g/kg/day in SO, with high‐quality protein accounting for > 50% and protein distributed evenly across meals to stimulate muscle protein synthesis and prevent and treat SO [46]. High‐protein diets commonly recommend protein intake of 25%–30% of total energy, which helps prevent excess carbohydrate intake, reduce lean mass loss, maintain appropriate protein nutritional status, and achieve better effects on weight loss and weight maintenance than calorie restriction alone [47]. However, for nondialysis patients with stage ≥ 3 CKD, protein intake should be individualized under guidance from nephrology or nutrition specialists and should not exceed 0.8 g/kg/day, to avoid increasing kidney burden.

A leucine‐rich diet may maximize the anabolic response while reducing overall dietary protein requirements [48]. This dietary pattern ensures adequate carbohydrate and fat intake, helps achieve weight‐loss goals, improves metabolic function, and maintains ADL [49]. For physically active older adults, a balanced leucine‐enriched amino acid formula may be considered. For example, whey protein is rich in leucine and other essential amino acids. When tolerated, an additional 15–20 g of whey protein within 1 h after resistance exercise may help prevent muscle decline and improve muscle strength and power in older adults [50]. Meta‐analyses show that HMB supplementation improves muscle mass and function in older adults with frailty and sarcopenia [51], and 3 g/day HMB is recommended for older adults with SO. Current evidence for creatine supplementation to enhance muscle gain during exercise intervention in older adults is limited. Although meta‐analyses are available [52, 53], the number of studies and sample sizes is limited, so routine additional creatine supplementation is not recommended. Vitamin D should be routinely supplemented in older adults to improve bone metabolism and immunoregulation [54, 55]. However, based on current evidence, routine vitamin D supplementation is not recommended as an effective measure to prevent or control sarcopenia [56].

### 6.2 Exercise Intervention

In older adults with obesity, exercise helps maintain or increase muscle mass and strength, protect bone health, reduce fat, improve physical function, and optimize metabolic health. Randomized controlled trials have shown that combining exercise with calorie restriction preserves muscle mass while improving insulin resistance and cardiometabolic risk factors [57]. A program with 150 min of moderate‐intensity exercise per week plus dietary intervention yields better body composition and functional outcomes than exercise or diet alone [58]. An exercise prescription centered on resistance training, supplemented with aerobic and balance exercises, is also more effective than aerobic or resistance training alone for improving cardiorespiratory fitness, muscle strength, and limiting muscle loss in older adults [59].

In older adults with obesity, exercise aims to safely preserve muscle mass, improve physical function, and reduce fall risk. Before starting a program, regular exercise habits, disease status, medications and disease‐related symptoms and signs should be assessed. Patients without a regular exercise habit or with symptoms/signs should start with low‐intensity exercise. Those who exercise regularly and have no symptoms/signs may begin with moderate‐intensity exercise. To refine the prescription, cardiopulmonary endurance, muscle strength and endurance, flexibility, balance, and gait should be evaluated. A combined program of aerobic, resistance, and balance training effectively supports obesity management in older adults. Exercise prescriptions should follow the FITT–VP framework and the principles of individualization, safety, and synergy.

Recommended exercise includes: low‐ to moderate‐intensity aerobic exercise (e.g., brisk walking, square dancing, or Baduanjin); resistance training (e.g., elastic bands, dumbbells, or fixed equipment); and balance training (e.g., single‐leg standing, lateral walking, or balance exercises) [60]. Aerobic exercise should be performed 3–5 days per week. During the first 4–6 weeks, the duration on each exercise day may be increased by 5–10 min every 1–2 weeks until reaching 30–60 min per day, either continuously or in accumulated bouts, for a total of 150–300 min per week. Resistance training should be performed 2–3 days per week; each movement should be repeated 6–20 times at maximal effort, with 2–4 sets per muscle group, 1–2 min between sets, and sessions on nonconsecutive days. Exercise intensity should moderately increase breathing and heart rate, with perceived exertion from light to moderate (increased breathing and heart rate during exercise, from easy to effortful), and physical capacity should recover by the next day. Performing 5–10 min of low‐intensity aerobic exercise and stretching before and after exercise helps prevent exercise‐related injury and promote recovery. During any adjustment to an exercise prescription, the individual's response should be monitored, with attention to adverse reactions caused by increased exercise volume, such as dyspnea, chest tightness, fatigue, and muscle soreness. If tolerance is poor, the exercise prescription should be adjusted promptly.

### 6.3 Pharmacological Treatment

In China, the lipase inhibitor orlistat and the nutrient‐stimulated hormone (NuSH) receptor agonists beinaglutide, liraglutide, semaglutide, tirzepatide, and mazdutide are approved for treating obesity in adults. Most NuSH registration studies have included few participants aged ≥ 65 years (weight‐loss studies of beinaglutide did not include people aged ≥ 65 years) and have often excluded those aged ≥ 80 years, as well as frail people or those with multiple comorbidities. A comprehensive assessment is recommended before starting treatment, and weight‐loss medications should be chosen based on evidence of benefit for specific complications or comorbidities, along with concurrent etiological and symptomatic treatment of these conditions.

#### 6.3.1 Lipase Inhibitors

Orlistat is a nonsystemic gastric and pancreatic lipase inhibitor. Small‐sample studies showed that oral orlistat 120 mg three times daily produced a placebo‐subtracted weight loss of 3%–5%, with similar effects in older and younger adults [61, 62, 63]. Fat‐soluble multivitamin supplementation is recommended for patients taking orlistat. Common adverse reactions include steatorrhea, increased stool frequency, and increased gastrointestinal gas. Orlistat is contraindicated in malabsorption syndrome, cholestasis, secondary obesity, organ transplantation, and patients taking cyclosporine.

#### 6.3.2 NuSH Receptor Agonists

Liraglutide 3.0 mg once daily for 56 weeks produced a placebo‐subtracted weight loss of 5.4% [64]. In overweight or obese patients with T2DM, 3.0 and 1.8 mg liraglutide for 56 weeks reduced weight by 4.0% and 2.7%, respectively [65]. Approximately 7% of participants were aged ≥ 65 years, and weight‐loss efficacy and safety did not differ significantly between older and younger patients. Among 4668 patients with T2DM (mean age, 64.2 years) treated with liraglutide 1.8 mg for 3.8 years, the risk of major adverse cardiovascular events (MACEs) decreased by 13% [66]. A post hoc analysis showed that in patients with T2DM aged ≥ 75 years, liraglutide also had favorable effects on time to first MACE, expanded composite outcomes, and all‐cause mortality risk [67].

Studies in patients with overweight or obesity showed that once‐weekly subcutaneous semaglutide 2.4 mg for 68 weeks produced placebo‐subtracted weight loss of 10.3%–17.4% [68, 69, 70]. The weight‐loss effect was attenuated in patients with T2DM (6.2%) [71]. Subgroup analyses suggested that the weight‐loss effect of semaglutide was independent of age [72]. In one study (*n* = 271; mean age, 56 years), semaglutide 2.4 mg for 68 weeks reduced body weight, significantly relieved obesity‐related knee osteoarthritis pain, and improved ADL [73]. Semaglutide 2.4 mg significantly reduced the risk of composite heart failure outcomes by 69% and improved heart failure symptoms in patients with obesity‐related heart failure with preserved ejection fraction [74]. In addition, a study (*n* = 8803; mean age, 61.6 years) showed that semaglutide 2.4 mg reduced MACE risk by 20% in patients with overweight or obesity and ASCVD, with similar cardiovascular benefits across age groups in subgroup analyses [75]. In a study of noncirrhotic nonalcoholic steatohepatitis (*n* = 534; mean age, 56.3 years), semaglutide 2.4 mg achieved resolution of metabolic dysfunction‐associated steatohepatitis and improvement of liver fibrosis in 62.9% and 36.8% of patients with MASLD, respectively, with no efficacy difference across age groups in subgroup analyses [76].

A pooled analysis of the SUSTAIN 1–5 studies showed that semaglutide had similar glucose‐lowering and weight‐loss effects in patients with T2DM aged ≥ 70 years and ≤ 50 years; 37%–59% and 40%–79% of older participants achieved > 5% weight loss with semaglutide 0.5 and 1.0 mg, respectively [77]. The SUSTAIN 6 study (*n* = 1648; mean age, 64.6 years) showed that semaglutide 0.5 and 1.0 mg reduced MACE risk by 26% in patients with T2DM and cardiovascular disease or high cardiovascular risk, with similar cardiovascular benefits in those aged ≥ 65 and < 65 years [78]. Results from the FLOW trial showed that, among patients with T2DM and CKD, including 1767 patients with heart failure at baseline, once‐weekly semaglutide 1.0 mg significantly reduced the risk of the major kidney composite endpoint by 24% [79]. The STRIDE study (*n* = 792; mean age, 68 years) showed that semaglutide 1.0 mg increased maximum walking distance by 13% in patients with T2DM and symptomatic peripheral arterial disease [80].

Tirzepatide is a dual glucose‐dependent insulinotropic polypeptide/glucagon‐like peptide‐1 receptor agonist. A series of clinical trials on tirzepatide for weight loss showed that once weekly subcutaneous tirzepatide 5, 10, and 15 mg produced significant dose‐dependent weight loss. After subtracting placebo effects, body weight decreased by 10%–20% in patients with overweight or obesity, with or without T2DM [81, 82, 83, 84, 85]. Subgroup analyses suggested that weight‐loss efficacy and safety in older patients (aged ≥ 65 years; *n* = 426) were similar to those in younger patients. The global phase III SURPASS program showed that once weekly tirzepatide 5, 10, and 15 mg reduced hemoglobin A1c (HbA1c) by 1.9%–2.1% and body weight by 7.0–9.5 kg after subtracting placebo effects [86], and reduced liver fat content by 8.09% [87]. Subgroup analyses showed that across different tirzepatide doses, older patients (aged ≥ 65 years; *n* = 1278) and younger patients similarly achieved the composite endpoint of HbA1c ≤ 6.5% and weight loss ≥ 10% without hypoglycemia [88]. A study (*n* = 13,299; mean age, 64.1 years) showed that tirzepatide reduced MACE risk by 8%, HbA1c by 1.7%, and body weight by 12.1% [89]. Clinical studies of tirzepatide for obesity with OSA showed that 10 or 15 mg reduced body weight and significantly improved OSA symptoms by reducing apnea and hypopnea and improving sleep quality [90].

Mazdutide is a dual glucagon/glucagon‐like peptide‐1 receptor agonist. In Chinese adults with overweight or obesity, once weekly mazdutide 4 and 6 mg for 32 weeks produced placebo‐subtracted weight loss of 10.5% and 13.0%, respectively. At 48 weeks, weight loss reached 11.3% and 14.3%, respectively, and liver fat content decreased by 30%–50% [91]. However, data in patients aged ≥ 65 years are limited, so these findings cannot yet be extrapolated to older adults. In Chinese patients with T2DM, mazdutide 4 and 6 mg monotherapy for 24 weeks reduced HbA1c by 1.4% and 2.0% and body weight by 4.4% and 6.2%, respectively, after subtracting placebo effects [92]. In patients with T2DM inadequately controlled by metformin, with or without sodium‐glucose cotransporter 2 inhibitors or sulfonylureas, add‐on mazdutide 4 and 6 mg for 28 weeks reduced HbA1c by 1.6% and 1.7% and body weight by 6.6% and 8.5%, respectively [93]. Across these two studies, 107 patients with T2DM aged ≥ 65 years received mazdutide, and no overall efficacy or safety differences were observed between older and younger patients.

The most common adverse reactions of NuSH receptor agonists are gastrointestinal symptoms such as nausea, diarrhea, and vomiting, and older patients have a higher proportion of more severe gastrointestinal adverse events. In older adults, rare adverse reactions such as gallstones and acute pancreatitis should also be considered. For older patients taking multiple medications, delayed gastric emptying and drug‐related gastrointestinal reactions, especially vomiting, may affect the absorption of other medications and require particular attention. In summary, NuSH receptor agonist weight‐loss medications also improve blood glucose, blood pressure, and lipid profiles. Some data have been accumulated on NuSH receptor agonists in older populations and evidence of benefit for ASCVD, CKD, peripheral arterial disease, metabolic dysfunction‐associated steatohepatitis, OSA, knee osteoarthritis, and other conditions. Therefore, NuSH receptor agonists may be considered for the management of obesity and related complications or comorbidities in older adults. Before treatment, clinicians should fully discuss risks and benefits with patients and families. Treatment should start at a low dose; the rate of weight loss should be reasonably controlled; and gastrointestinal adverse reactions as well as changes in muscle mass and muscle strength should be closely monitored to avoid malnutrition due to gastrointestinal adverse reactions and muscle loss due to weight reduction [94]. When older adults with obesity have T2DM, cardiovascular or renal disease, lower‐extremity vascular disease, or OSA, timely combination therapy with NuSH receptor agonists with evidence of benefit for these diseases is recommended.

### 6.4 Cognitive Behavioral Therapy

Evidence supporting cognitive behavioral therapy (CBT) in older adults with obesity remains insufficient; relevant approaches include education, motivational interviewing, self‐monitoring, goal setting, and individualized feedback [95]. Strategies may be adapted to age‐related cognitive decline, such as simplified goal setting, visual feedback tools, and strengthened family or caregiver involvement, while considering factors that may affect adherence to CBT in older adults, including social isolation, depression, and disease burden [96]. Therefore, CBT should prioritize health as the main goal, focusing on improvement of comorbidities and quality of life rather than weight loss alone. Continuous support, regular follow‐up, and enhanced family or social participation are essential for improving adherence, long‐term management, and prevention of weight regain.

### 6.5 Bariatric Surgery

Although systematic reviews suggest that denying bariatric surgery solely because of age is unreasonable [97, 98], an obesity paradox exists in older adults when BMI is used as the reference indicator. In addition, weight loss in older adults with obesity should be slow rather than rapid and modest rather than excessive. Therefore, bariatric surgery in older adults with obesity should be performed cautiously only after considering health status, frailty, comorbidities and their treatment; thoroughly assessing major organ function and surgical tolerance; weighing benefits and risks through multidisciplinary evaluation; and obtaining informed consent [99].

### 6.6 Follow‐Up

Timely monitoring and follow‐up are essential during treatment of older adults with obesity. Patients are advised to monitor body weight weekly, and wearable smart devices may also be used for follow‐up. When weight‐loss medication is initiated for obesity‐related complications or comorbidities, drug safety and efficacy should be assessed at least once per month during the first 3 months. Thereafter, follow‐up frequency may be adjusted according to the individual patient, but body fat, visceral fat, muscle mass and function, and metabolic indicators should still be evaluated every 3 months. If weight loss is < 5% after 6–12 months and complications do not improve, clinicians should consider whether the treatment plan is inappropriate or whether other factors are affecting weight reduction. In patients with unintentional weight loss of > 5% over a short period, other causes of rapid weight loss should be considered. With the increasing use of telemedicine and wearable devices, app‐based dietary records, remote exercise guidance, and wearable monitoring of heart rate and activity can complement traditional follow‐up, especially for older adults with obesity who have limited mobility or live in remote areas, and may improve treatment adherence and long‐term management.

## 7 Part VI Summary

Obesity management in older adults differs substantially from that in younger people because of age‐related physiological decline, comorbidities, polypharmacy, and specific nutritional needs. Clinical care should start with a comprehensive assessment of overall health, sarcopenia, obesity‐related complications, and comorbidities, with careful weighing of the benefits and risks of weight loss. Lifestyle intervention with diet and exercise is the first‐line approach. For patients with metabolic diseases, ASCVD, OSA, knee osteoarthritis, or related conditions, weight‐loss drugs with proven benefits for these diseases are recommended, alongside etiological and symptomatic treatment of comorbidities. Further randomized controlled trials in older adults with obesity are required to support scientific, rational, standardized, and individualized management strategies.

**Expert Panel Composition (Sorted by Pinyin of Last Names)**.

**Guiding Experts**.

Ji Fusui (Beijing Hospital, National Center for Gerontology; National Clinical Research Center for Gerontology; The Key Laboratory of Geriatrics of NHC; Institute of Geriatric Medicine, Chinese Academy of Medical Sciences).

Wu Jing (Chinese Center for Disease Control and Prevention, National Center for Chronic and Noncommunicable Disease Control and Prevention).

**Corresponding Authors**.

Pan Qi (Beijing Hospital, National Center for Gerontology; National Clinical Research Center for Gerontology; The Key Laboratory of Geriatrics of NHC; Institute of Geriatric Medicine, Chinese Academy of Medical Sciences).

Guo Lixin (Beijing Hospital, National Center for Gerontology; National Clinical Research Center for Gerontology; The Key Laboratory of Geriatrics of NHC; Institute of Geriatric Medicine, Chinese Academy of Medical Sciences).

**Expert Committee Members**.

Bi Yan (Nanjing Drum Tower Hospital, The Affiliated Hospital of Nanjing University Medical School).

Zeng Tianshu (Union Hospital, Tongji Medical College, Huazhong University of Science and Technology).

Chang Cuiqing (Peking University Third Hospital).

Chen Liming (Tianjin Medical University Zhu Xianyi Memorial Hospital).

Chen Wei (Peking Union Medical College Hospital, Chinese Academy of Medical Sciences).

Chen Xiaoping (China‐Japan Friendship Hospital).

Dou Jingtao (The First Medical Center of Chinese PLA General Hospital).

Gao Xiang (Institute of Nutrition/Institute of Clinical Sciences, Fudan University).

Guan Haixia (Guangdong Provincial People's Hospital).

Guo Lixin (Beijing Hospital, National Center for Gerontology; National Clinical Research Center for Gerontology; The Key Laboratory of Geriatrics of NHC; Institute of Geriatric Medicine, Chinese Academy of Medical Sciences).

Guo Jianjun (Capital University of Physical Education and Sports).

Jiang Sheng (The First Affiliated Hospital of Xinjiang Medical University).

Kuang Hongyu (The First Affiliated Hospital of Harbin Medical University).

Li Chunlin (The Second Medical Center of Chinese PLA General Hospital).

Li Sheyu (West China Hospital, Sichuan University).

Li Xia (The Second Xiangya Hospital of Central South University).

Li Yiming (Huashan Hospital, Fudan University).

Liang Yuzhen (The Second Affiliated Hospital of Guangxi Medical University).

Lin Xiahong (The Seventh Affiliated Hospital of Sun Yat‐sen University).

Liu Shiwei (Shanxi Bethune Hospital).

Lu Bin (Huadong Hospital, Fudan University).

Ma Jing (Renji Hospital, Shanghai Jiao Tong University School of Medicine).

Pan Qi (Beijing Hospital, National Center for Gerontology; National Clinical Research Center for Gerontology; The Key Laboratory of Geriatrics of NHC; Institute of Geriatric Medicine, Chinese Academy of Medical Sciences).

Qin Guijun (The First Affiliated Hospital of Zhengzhou University).

Qin Yingfen (The First Affiliated Hospital of Guangxi Medical University).

Quan Jinxing (Gansu Provincial Hospital).

Ran Xingwu (West China Hospital, Sichuan University).

Song Jinghai (Beijing Hospital, National Center for Gerontology; National Clinical Research Center for Gerontology; The Key Laboratory of Geriatrics of NHC; Institute of Geriatric Medicine, Chinese Academy of Medical Sciences).

Sun Ke (Beijing Hospital, National Center for Gerontology; National Clinical Research Center for Gerontology; The Key Laboratory of Geriatrics of NHC; Institute of Geriatric Medicine, Chinese Academy of Medical Sciences).

Sun Yu (Xuanwu Hospital, Capital Medical University).

Wang Haining (Peking University Third Hospital).

Wang Ningjian (Pudong Gongli Hospital, Shanghai University of Medicine & Health Sciences).

Wang Zhengzhen (Beijing Sport University).

Wu Hao (Capital Medical University).

Xiao Xinhua (The First Affiliated Hospital of University of South China).

Xu Jixiong (The First Affiliated Hospital of Nanchang University).

Xu Jing (The Second Affiliated Hospital of Xi'an Jiaotong University).

Xu Yong (The Affiliated Hospital of Southwest Medical University).

Xu Yushan (The First Affiliated Hospital of Kunming Medical University).

Yang Ying (The Affiliated Hospital of Yunnan University).

Yu Dongni (Beijing Hospital, National Center for Gerontology; National Clinical Research Center for Gerontology; The Key Laboratory of Geriatrics of NHC; Institute of Geriatric Medicine, Chinese Academy of Medical Sciences).

Yuan Mingxia (Beijing Friendship Hospital, Capital Medical University).

Zang Li (The First Medical Center of Chinese PLA General Hospital).

Zhang Cuntai (Tongji Hospital, Tongji Medical College, Huazhong University of Science and Technology).

Zhang Huijie (Zhongshan Hospital, Fudan University).

Zhang Qiu (The First Affiliated Hospital of Anhui Medical University).

Zheng Xin (China Rehabilitation Research Center, Beijing Bo'ai Hospital).

**Lead Writers/Principal Authors**.

Zhang Jie (Beijing Hospital, National Center for Gerontology; National Clinical Research Center for Gerontology; The Key Laboratory of Geriatrics of NHC; Institute of Geriatric Medicine, Chinese Academy of Medical Sciences).

Li Sheyu (West China Hospital, Sichuan University).

**Writing Secretariat Members**.

Man Fuli (Beijing Hospital, National Center for Gerontology; National Clinical Research Center for Gerontology; The Key Laboratory of Geriatrics of NHC; Institute of Geriatric Medicine, Chinese Academy of Medical Sciences).

Liu Xiaochuan (Beijing Hospital, National Center for Gerontology; National Clinical Research Center for Gerontology; The Key Laboratory of Geriatrics of NHC; Institute of Geriatric Medicine, Chinese Academy of Medical Sciences).

Fei Sijia (Beijing Hospital, National Center for Gerontology; National Clinical Research Center for Gerontology; The Key Laboratory of Geriatrics of NHC; Institute of Geriatric Medicine, Chinese Academy of Medical Sciences).

Jiang Yuanyuan (Beijing Hospital, National Center for Gerontology; National Clinical Research Center for Gerontology; The Key Laboratory of Geriatrics of NHC; Institute of Geriatric Medicine, Chinese Academy of Medical Sciences).

## Author Contributions

Professors Lixin Guo and Qi Pan conceptualized and led the framework development, established the working group, and reviewed the final draft. Professors Fusui Ji, Jing Wu directed the research design and provided academic guidance for the consensus. Professors Zhang jie, Li Sheyu drafted the initial manuscript, collected and analyzed the data, and revised the full text critically. Professors Guo Lixin and Pan Qi organized the multidisciplinary expert panel, facilitated group discussions, secured research funding, and provided administrative and technical support to finalize the consensus document. Professors Yan Bi, Cuiqing Chang, Liming Chen, Wei Chen, Xiaoping Chen, Jingtao Dou, Xiang Gao, Haixia Guan, Jianjun Guo, Sheng Jiang, Hongyu Kuang, Chunlin Li, Sheyu Li, Xia Li, Yiming Li, Yuzhen Liang, Xiahong Lin, Shiwei Liu, Jing Ma, Guijun Qin, Yingfen Qin, Jinxing Quan, Xingwu Ran, Jinghai Song, Ke Sun, Yu Sun, Haining Wang, Ningjian Wang, Zhengzhen Wang, Hao Wu, Xinhua Xiao, Jixiong Xu, Jing Xu, Yong Xu, Xu Yushan, Ying Yang, Mingxia Yuan, Li Zang, Tianshu Zeng, Cuntai Zhang, Huijie Zhang, Qiu Zhang, Xin Zheng contributed to data analysis, interpretation, and critical revision of the intellectual content. Fuli Man provided data collection, administrative and technical support, and assisted in manuscript proofreading. All authors reviewed, revised, and approved the final consensus for publication.

## Funding

This work was supported by the National High Level Hospital Clinical Research Funding (BJ‐2025‐209), Capital's Funds for Health Improvement and Research (2024‐1‐4053), and National High Level Hospital Clinical Research Funding (BJ‐2024‐144).

## Disclosure

Practice Guideline Registration: Practice Guideline Registration for Transparency (PREPARE‐2025CN1576).

## Conflicts of Interest

Lixin Guo is an Editorial Board Member of Aging Medicine and a co‐author of this article. To minimize bias, they were excluded from all editorial decision‐making related to the acceptance of this article for publication.

## Data Availability

Data sharing is not applicable to this article as no datasets were generated or analyzed during the current study.
